# Development and psychometric testing of a new measure of the determinants that influence the adoption of WhatsApp in hospitals

**DOI:** 10.1371/journal.pone.0262003

**Published:** 2021-12-30

**Authors:** Anna De Benedictis, Emanuele Lettieri, Michela Piredda, Raffaella Gualandi, Maddalena De Maria, Daniela Tartaglini

**Affiliations:** 1 Department of Healthcare Professions, Campus Bio-Medico of Rome University Hospital, Rome, Italy; 2 Department of Economics, Management and Industrial Engineering, Politecnico of Milan, Milan, Italy; 3 Research Unit Nursing Science, Campus Bio-Medico of Rome University, Rome, Italy; 4 Department of Biomedicine and Prevention, University of Rome “Tor Vergata”, Rome, Italy; Universita degli Studi di Udine, ITALY

## Abstract

**Background:**

Healthcare contexts are witnessing a growing use of applications to support clinical processes and to communicate between peers and with patients. An increasing number of hospital professionals use instant-messaging applications such as WhatsApp in their daily work. Previous research has mainly focused on the advantages and risks of WhatsApp usage in different clinical settings, but limited evidence is available about whether and how individual and organizational determinants can influence the use of WhatsApp in hospitals. Moreover, instruments to explore this phenomenon are lacking. A theoretical four-factor model based on the ‘Technology Acceptance Model’ and the Institutional Theory, guided the development of a new measure of the individual and institutional determinants of WhatsApp usage in hospitals.

**Aim:**

To develop and psychometrically test the questionnaire ‘Digital Innovation Adoption in Hospitals’.

**Method:**

A panel of researchers and clinical experts generated an initial pool of 35 items by identifying and adapting items from existing measures. These items were assessed for content and face validity by fourteen experts. The final 28-item ‘Digital Innovation Adoption in Hospitals’ questionnaire comprising four sections (Perceived risks, Perceived usefulness, Regulative factors and Normative factors) was administered online to nurses and physicians. Construct validity was tested through confirmatory factor analysis.

**Results:**

The sample included 326 hospital nurses and physicians. The theoretical four-factors model was confirmed and the confirmatory factor analysis yielded acceptable fit indexes. The correlations between the factors were significant and ranged from -0.284 to 0.543 (p < .01). Reliability in terms of internal consistency was satisfactory with Cronbach’s alpha coefficient ranging from 0.918–0.973.

**Conclusion:**

This study is the first to provide a validated tool to evaluate the use of WhatsApp in hospitals. The new instrument shows reasonable psychometric properties and is a promising and widely applicable measure of factors that influence the use of WhatsApp in hospitals.

## Introduction

In the healthcare context and particularly in hospitals, a growing use of mobile health applications to support clinical and care processes is documented [1–6]. An increasing number of hospital professionals use instant-messaging applications such as WhatsApp in their daily work and to communicate between peers and with patients [1–9]. The literature highlighted both advantages and possible risks [10, 11] related to the use of WhatsApp in healthcare in different clinical and care settings. For example, WhatsApp is perceived effective in facilitate communication among physicians, in the discussion of clinical cases or sharing interest or knowledge in groups [10], in medical education [12], for some kind of teleconsultation [2], or to improve the adherence to care and the health outcomes for specific clinical areas [13]. Recent research is seeking to evaluate the impact of WhatsApp on the quality and safety of patient care [11], or is focused on the usefulness of WhatsApp in different clinical and care settings [6–11, 14–28]. However, there are still limited studies exploring whether and how individual and organizational determinants can lead the adoption of WhatsApp in hospitals between peer and patients [29]; moreover, instruments that can help to explore this phenomenon are lacking. For this reasons this study aims to develop a new measure of the individual and institutional determinants that influence the use of WhatsApp in hospitals, as perceived by nurses and physicians.

Two well-established theories can be considered to explore the determinants of WhatsApp adoption in hospitals: the Technology Acceptance Model (TAM) [30, 31] and the Institutional theory [32–34]. The TAM [32, 33] is the most widely-used rational model to explore what leads people to accept or reject Information Technology. It identifies two main antecedents, perceived usefulness and perceived ease of use of technology. Perceived usefulness measures ‘the degree to which a person believes that using a particular system would enhance his or her job performance’ [30], and ease of use measures ‘the degree to which a person believes that using a system would be free of effort’ [30, 31]. Institutional theory, meanwhile, is based on the assumption that individual behaviours are modelled by regulations, social norms and meaning systems, and that institutions embodied in routines rely on the automatic cognition and uncritical processing of existing schemata, and privilege consistency with stereotypes and speed over accuracy. According to this perspective, individuals are embedded in institutional pillars that limit the scope of their rational assessments and direct the engagement of specific behaviours [32–34]. Scott [32–34] defines the three institutional pillars as follows: the regulative pillar (which concerns the existence of regulations, rules and processes whose breach is monitored and sanctioned), the normative pillar (which introduces a social dimension of appropriate behaviours in the organisation) and the cultural pillar (which emphasizes the use of common schemas, frames and other shared symbolic representations that create attachment to the ‘appropriate’ behaviour).

In this study we focused on WhatsApp because it is one of the most widespread text messaging application used by an increasing number of healthcare professionals in their daily work and for different reasons [1–11, 14–28], and because its usage in hospitals is currently highly controversial. In fact, its ease of use–and penetration in our daily life–matches with the risks related to errors, communication misunderstandings and data protection.

## Aims

The study aims at developing and psychometrically testing the questionnaire ‘Digital Innovation Adoption in Hospitals’ (DIAH), a measure of the individual and institutional factors that influence the use of WhatsApp in hospitals as perceived by nurses and physicians.

## Material and methods

### Design

This was a cross-sectional questionnaire development and validation study following the guidelines of the European Statistical System for instrument development and validation [35]. These included five steps: (1) conceptualisation, (2) questionnaire design, (3) questionnaire testing, (4) revision and (5) data collection. These will be described in detail as follows.

### Conceptualisation and questionnaire design

**Fig 1:**
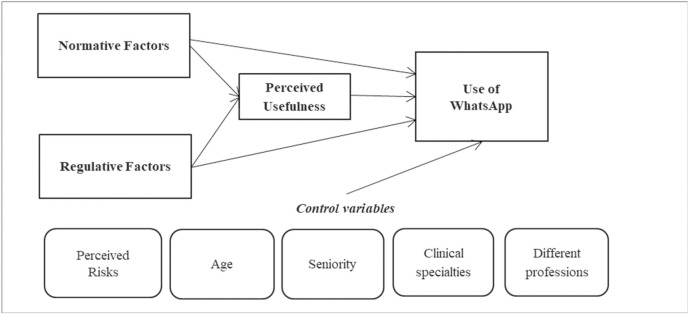
Theoretical framework.

A theoretical model (Fig 1) based on the TAM and the Institutional Theory, guided the instrument development [29]. Among the individual variables in our theoretical model we decided to include only ‘perceived usefulness’, excluding ‘ease of use’, because preliminary interviews and past experiences showed that physicians and nurses in our setting use smartphones and WhatsApp daily, so we could exclude problems related to the digital divide. Finally, some variables have been included as they are considered significant for a better understanding of the phenomenon [29]. To identify key concepts related to individual and institutional factors that influence the use of WhatsApp in hospitals, a comprehensive literature search was conducted in the PubMed and CINAHL databases, searching for articles published from inception to April 2019. This search was performed using keywords such as ‘WhatsApp’, ‘questionnaire’ and ‘synonyms’. Additional studies were identified through a hand search of reference lists and the PubMed instrument ‘similar articles’. Key concepts from the records retrieved were considered according to the theoretical model to generate a pool of items. The criteria for item creation were ‘ability to investigate intention to use digital innovation in hospitals by healthcare professionals’ and ‘appropriateness for the Italian context’. Based on the theoretical model [29], the scale for the measurement of perceived usefulness has been adapted from the studies of Venkatesh [36–39], and the scale for the measurement of normative and regulative factors has been adapted from the study of Scott [34]. The items were translated into the Italian language and cross-culturally adapted by a panel of experts.

### Questionnaire testing and revision

#### Face validity

The initial item pool comprised 35 indicators that were reviewed for face validity by a panel of four experts. The panel members were one nurse and one physician with more than 9 years of work experience, and two engineers with expertise in Information Science. Panel members were asked to evaluate each statement for clarity, ease of use and appropriateness [40]. Based on their comments and suggestions, two items were removed and changes were made in the wording of several items to increase clarity. The two removed items were related to the perceived usefulness of WhatsApp: "I use WhatsApp to share scientific information with my colleagues” and “I use WhatsApp for manage and share the agenda with my colleagues". The experts considered these items not appropriate and consistent with the aims of the study.

#### Content validity

The 33-item draft questionnaire was tested for content validity by 10 experts (including two nurses, three head nurses, two managers and three physicians) not involved in the preceding phase. They were individually interviewed using a semi-structured grid in a designated room by three researchers: one acted as interviewer, the other two helped with audio-recording and with filling out the grid for item evaluation. The semi-structured grid was created by researchers for this study, and included for each item the request to give an opinion regarding: clarity in content and language, utility, consistency with the research questions, and ease of reply. The experts were also asked to share any additional comments that they believed were important. The interviews were audio-recorded and lasted about 60 minutes on average. Experts were also asked to identify each item’s ability to measure the determinants of the intention to use WhatsApp in hospitals. The data collected were entered into a database and the themes that emerged from the interviews for each item were coded and analyzed. Based on the experts’ evaluation, five items were eliminated and five items were reworded. Two removed items were related to the perceived risks: “The use of WhatsApp to communicate patient data with other health professionals is safe and does not entail risks” and “The use of WhatsApp for communication between patients and health professionals is safe and does not involve risks”; the Experts considered them useless because very similar to other items. The other three items were evaluated not consistent with the aims of the study or very similar to other items: “My colleagues are using WhatsApp for personal reasons”; “The use of WhatsApp has the limit of the need for Internet connection; “The evaluation of images or videos sent via WhatsApp is not sufficient to make a diagnosis”.

#### Instrument

The final 28-item questionnaire was called ‘Digital Innovation Adoption in Hospitals’ (DIAH) and consisted of four main sections: ‘Perceived Risks’ (10 items), ‘Perceived Usefulness’ (9 items), ‘Regulative Factors’ (3 items) and ‘Normative Factors’ (6 items). The survey items are shown in Table 1. All questionnaire items were explored using a 7-point Likert scale from 1 = totally disagree, to 7 = totally agree. Data on demographic characteristic of participants were also gathered.

**Table 1:** Digital Innovation Adoption in Hospitals (DIAH) questionnaire [29].

Variables	Items/indicators				
**Control variables and characteristics of the respondent**					
General information	Age				
	Gender				
	Profession				
	Clinical Area or Unit				
	Academic role in this healthcare company				
	Work experience (indicate the number of years)				
	Work experience in this healthcare company (indicate the number of years)				
**Section 1: Perceived risks (PR)** *		**Mean**	**SD**	**Skew**	**Kurt**
	PR1. Sending clinical data via WhatsApp involves risks for health professionals	4.68	1.540	-.457	-.287
	PR2. The use of WhatsApp involves risks related to privacy and data protection	5.14	1.508	-.864	.349
	PR3. The use of WhatsApp carries the risk of uncontrolled spread of sensitive data	5.15	1.569	-.814	.141
	PR4. To communicate through WhatsApp involves clinical risks as it is not documented within the medical record	5.28	1.603	-.976	.488
	PR5. The use of WhatsApp for communication can generate misunderstandings with the patient	4.87	1.507	-.740	.269
	PR6. Sending clinical-care data via WhatsApp involves risks for the patient	4.64	1.504	-.471	-.214
	PR7. The use of WhatsApp involves the risk of incorrect clinical evaluations	4.90	1.524	-.673	.084
	PR8. The use of WhatsApp involves the risk of incorrect diagnosis and clinical decisions	4.74	1.610	-.568	-.278
	PR9. The use of WhatsApp involves the risk of compromising the patient-physicians relationship	4.40	1.726	-.258	-.794
	PR10. The use of WhatsApp for the transmission of sensitive patient data should be subject to consent by the patient for personal data handling	5.44	1.511	-1.010	.724
**Section 2: Perceived Usefulness (PU)** *		**Mean**	**SD**	**Skew**	**Kurt**
Individual determinants: Perceived Usefulness	PU1. I am convinced that the use of WhatsApp improves communication	4.47	1.534	-.435	-.391
	PU2. Using WhatsApp lets you know if the messages have been read by colleagues	4.75	1.509	-.722	.258
	PU3. To use WhatsApp for work is time saving because it is faster than phone or mail	4.67	1.610	-.709	-.032
	PU4. I am convinced that if everyone used WhatsApp, there would be greater and more effective sharing of clinical knowledge	3.85	1.686	-.102	-.760
	PU5. The use of WhatsApp positively affects my research activity (e.g. it is easier to share data and results)	4.14	1.595	-.301	-.461
	PU6. The use of WhatsApp positively affects my teaching activity	4.07	1.517	-.269	-.254
	PU7. Using WhatsApp to monitor patients’ clinical conditions increases the likelihood of improvement of their clinical situation	4.15	1.724	-.314	-.718
	PU8. Use of WhatsApp facilitates the doctor-patient relationship	4.08	1.626	-.407	-.553
	PU9. Using WhatsApp in my work allows me to effectively exchange information with the patient, thus avoiding a medical examination	2.89	1.729	.544	-.774
**Section 3: Regulative Factors (RF)** *		**Mean**	**SD**	**Skew**	**Kurt**
Organisational determinants: Regulative Factors	RF1. The Hospital Management asks me not to use WhatsApp among colleagues	3.26	1.681	.249	-.617
	RF2. The Hospital Management asks me not to use WhatsApp with patients	4.30	1.806	-.270	-.700
	RF3. The Hospital Management asks me not to communicate sensitive patient data via WhatsApp	5.00	1.727	-.677	-.131
**Section 4: Normative Factors (NF)** *		**Mean**	**SD**	**Skew**	**Kurt**
	NF1. My colleagues are using WhatsApp for professional reasons	4.55	1.499	-.533	.244
	NF2. My colleagues are using WhatsApp to share scientific information	4.62	1.514	-.547	.256
	NF3. My colleagues are using WA to communicate patient information	3.75	1.696	-.181	-.746
	NF4. My patients ask me to use WhatsApp	3.42	1.799	.039	-1.048
	NF5. My patients prefer doctors who use WhatsApp	3.52	1.603	-.037	-.324
	NF6. My patients are more likely to recover if they are using WhatsApp for care continuity	3.19	1.626	.096	-.674

* PR = Perceived Risks; PU = Perceived Usefulness; RF = Regulative Factors; NF = Normative Factors.

### Data collection

#### Setting and participants

Hospitals are exemplary settings for the study, since past research strongly supports both TAM-related and institutional explanations [41] and a hospital offered an ideal setting for investigating whether and how the two theories are correlated. Specifically, the Italian Hospital involved in the study is a medium-sized (around 300 beds), multi-disciplinary teaching hospital and it is home to a whole range of clinical, teaching and research activities. The choice of a single case study offers the opportunity to eliminate potentially confusing factors due to the heterogeneity that different hospitals might show.

The questionnaire was deposited online using a Google module. The principal investigator sent the link for the online questionnaire through e-mail to all nurses (n = 380) and to 250 physicians representing different clinical areas of a medium-size university hospital in Rome between February and September 2020. Three monthly reminders were sent through e-mails by the principal investigator. To test dimensionality the sample size recommended is of 10 participants for each item of the scale [42], therefore, a minimum of 280 participants was sought.

### Ethics statement

The study was approved by the University Campus Bio-Medico Ethics Board, (Approval number: 61/16 OSS ComEt), and was conducted in accordance with the principles of the Declaration of Helsinki developed in Brazil [43]. Health professionals were invited to participate through an information letter including the purpose and procedures of the study, and an informed consent was granted by professionals involved in the study. Data were collected anonymously and made visible only by the principal investigator.

### Statistical analyses

Descriptive statistics of means, frequencies and percentages for the sample’s demographic characteristics and items were performed. Since the factor structure of ‘Digital Innovation Adoption in Hospitals’ (DIAH) was based on a theoretical model [29], we used a confirmatory approach to evaluate its construct validity. As suggested by expert reviewers, the Bartlett’s test and the Kaiser-Meyer-Olkin (KMO) index were calculated. The Bartlett’s test must be significant, and values ≥ 0.90 of KMO are considered excellent; 0.80–0.90 good; 0.70–0.80; moderate; 0.60–0.70 acceptable and ≤ 0.60 not-acceptable. A multifaceted approach was used to evaluate the goodness of model fit with the following indices: the Root Mean Square Error of Approximation (RMSEA; values ≤ 0.06 indicate a good fit); the Comparative Fit Index (CFI; values > 0.90 indicate a good fit); the Tucker and Lewis Index (TLI; values > 0.90 indicate a good fit); the Standardized Root Mean Square Residual (SRMR; values ≤ 0.08 indicate a good fit) [38–40]. The traditional chi-square test (χ^2^) was also computed, but not used in interpreting the model fit because it is influenced by sample size. Internal consistency was evaluated thorough Cronbach’s Alpha coefficients (α ≥ 0.90 were considered excellent; 0.8 ≤ α < 0.9 good; 0.7 ≤ α < 0.8 acceptable; 0.6 ≤ α < 0.7 questionable; 0.5 ≤ α < 0.6 poor; α < 0.5 unacceptable) [44], and Factor score determinacy coefficients (values > 0.70 were considered as adequate) [45]. The difference between scores of factors for participant socio-demographic variables was analyzed through t-test or ANOVA when appropriate. Generalized Linear Models (GLMs) were used to adjust for correlated socio-demographic variables. Significance was set at p < 0.05. Statistical analyses were performed using SPSS 21.0 and MPLUS 6.12 [44, 45].

## Results

### Sample and item descriptive characteristics

The final sample consists of 326 healthcare professionals including 220 (67.5%) nurses and 106 (32.5%) physicians, with a response rate of 52%. There were 97 (30%) men with mean age of 37.4 years (range 22–69, SD 9.8) and a mean work experience of 17.1 (range 0.3–45, SD 9) years. Detailed socio-demographic data of participants are described in Table 2.

**Table 2:** Characteristics of participants (n = 326).

	N (%)
Age	37.4 (8.9)
Work experience (years)	12.4 (9.4)
Women	229 (70.0)
Qualification	
Physician	106 (32.5)
Nurse	220 (67.5)
Clinical area	
Outpatient	28 (8.6)
Surgical	49 (15.0)
Medical	134 (41.0)
Critical care/emergency	50 (15.2)
Services^§^	35 (10.7)
Operating theatre	18 (5.5)
Management^¶^	12 (3.7)

^§^ Services include for instance: Endoscopy, Haemodynamics, Radiology;.

^¶^ Management include: Quality assurance, Infection control, Risk management, Case managers.

### Construct validity

**Fig 2:**
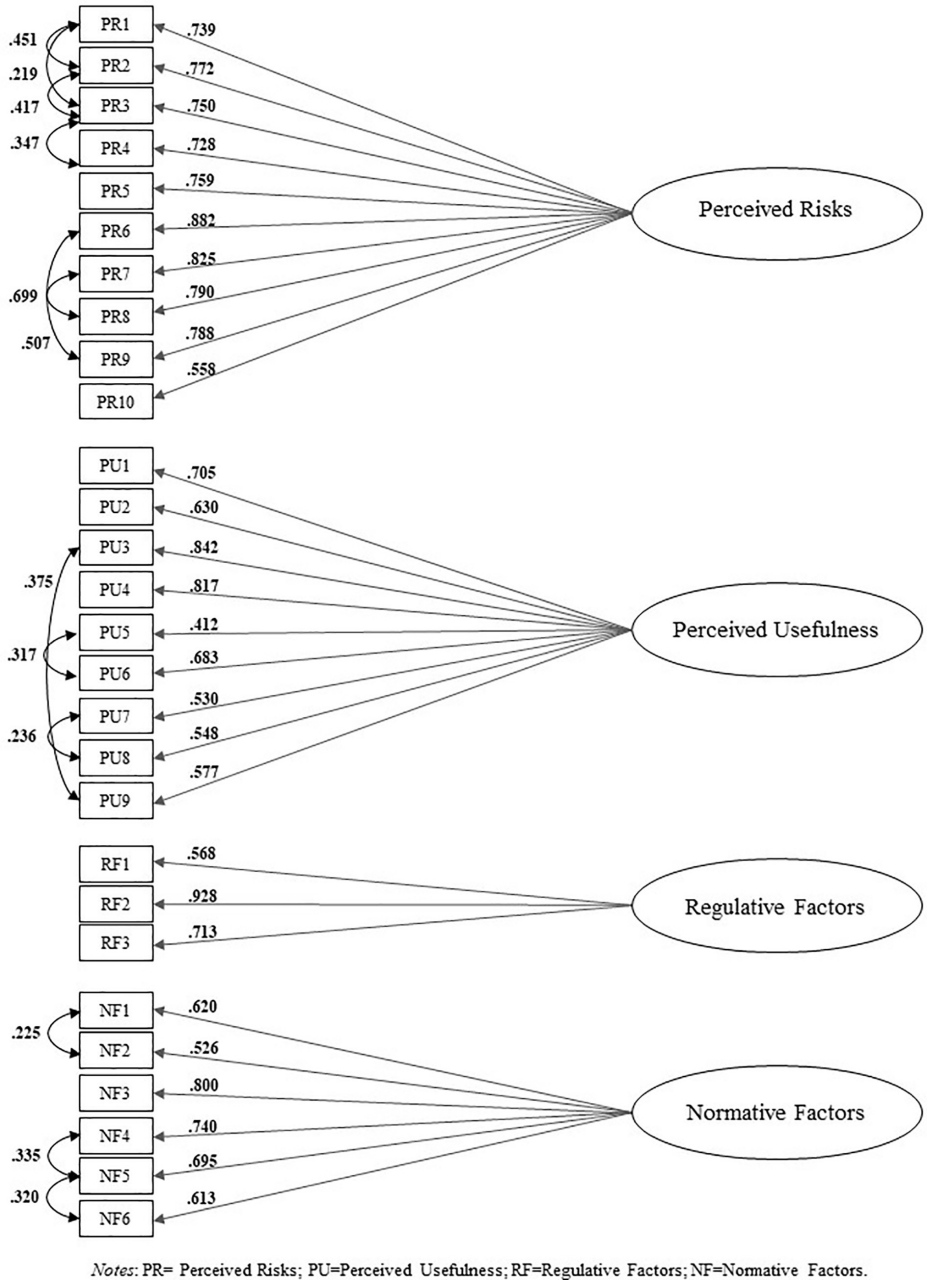
Model of ‘Digital Innovation Adoption in Hospitals’ (DIAH) factors at confirmatory factor analysis.

The data set was considered suitable for factor analysis because the KMO was 0.886 and Bartlett’s test of sphericity was significant (p < .001). The confirmatory factor analysis (CFAs) was performed using a maximum likelihood robust (MLr) estimator to account for the non-normality of item distribution [45]. The model with four factors (PR = *Perceived Risks*, PU = *Perceived Usefulness*, RF *= Regulative Factors*, NF *= Normative Factors)* yielded the following fit indices: χ2(344, N = 326) = 1125.664, p < .001; CFI = 0.915; TLI = 0.895; RMSEA = 0.083 (C.I. 90% 0.078–0.89, Probability RMSEA < = .05, p< .0001); CFI = 0.814; TLI = 0.796; SRMR = 0.067. The loadings were all higher than 0.4 and significant at p< .001. Evaluation of the modification indices showed that the misfit was due to excessive covariance between several items: PR1 and PR2; PR1 and PR3; PR2 and PR3; PR3 and PR4; PR6 and PR9; PR7 and PR8; PU5 and PU6; PU3 and PU9; PU7 and PU8; NF1 and NF2; NF5 and NF6; NF4 and NF5 (Table 1). Specification of these covariances was theoretically supported because of proximity and similar wording of items that seemed to increase the shared area of meaning [46]. In particular, with respect to the factors ‘Perceived Risks’, the items PR1, PR2, PR3 and PR4 (Fig 2) reflect the proximity of the items referring to perceived risk for patients and operators regarding privacy; PR6 and PR9 are related to connections between patients’ perceived risks about data protection and impairment of the doctor-patient relationship. The items PR7 and PR8 concern perceived risk due to improper use of WhatsApp. With respect to ‘Perceived Usefulness’ the shared covariance is due to proximity of items. In particular, PU5 and PU6 explore how the use of WhatsApp affects one’s professional activity (with a focus on research and teaching); PU3 and PU9 refer to the impact of WhatsApp on time saving; and PU7 and PU8 relate to remote monitoring and the doctor-patient relationship. Finally, several items of ‘Normative Factors’ presented correlation between residuals, in particular NF1 and NF2, both concerning the use of WhatsApp for professional reasons; NF4 and NF5, relating to patients’ preference of the use of WhatsApp for clinical-care reasons; NF5 and NF6 showed correlated residuals that can be explained because the clinicians who believe patients prefer professionals who use WhatsApp, also believe that its use for continuity of care can increase patient recovery. This model was therefore specified yielding the following acceptable fit indices: χ^2^(332, N = 326) = 703.692, p < .0001; RMSEA = 0.059 (C.I. 90% 0.053–0.065), probability RMSEA < = 0.05; p = .010; CFI = 0.912; TLI = 0.900; SRMR = 0.062, p < .0001. All factor loadings were higher than 0.4 and significant at p < .001 (Fig 2).

### Reliability, factor scores and correlations

The Cronbach’s alpha coefficient (α) for ‘Regulative Factors’ was α = 0.770, for ‘Perceived Usefulness’ was α = 0.863, for ‘Perceived Risks’ α = 0.936, and for ‘Normative Factors’ α = 0.843. The factor score determinacy coefficients were 0.942 for Regulative Factors’, 0.954 for ‘Perceived Usefulness’, 0.973 for ‘Perceived Risks’, and 0.918 for ‘Normative Factors’ (Table 3). The mean score and standard deviation M (±) for the items of ‘Regulative Factors’ was M = 4.18 (±1.43), for ‘Perceived Usefulness’ M = 4.11 (±1.11), for ‘Perceived Risks’ M = 4.92 (±1.24), and for ‘Normative Factors’ M = 3.84 (±1.21), all on a range 1–7 (Table 3). The correlations between the four sections were all significant at p < .01 and ranged from—.284 to .543. In particular, the correlation was positive and high between ‘Normative Factors’ and ‘Perceived Usefulness’ (r = .543), and between ‘Regulative Factors’ and ‘Perceived Risks’ (r = .306), and negative between ‘Regulative Factors’ and ‘Perceived Usefulness’ (r = —.219), as well as between ‘Perceived Usefulness’ and ‘Perceived Risks’ (r = —.284), ‘Normative Factors’ and ‘Perceived Risks’ (r = .310) and ‘Regulative Factors’ and ‘Normative Factors’ (r = —.296) (Table 3).

**Table 3:** Scores, reliability and correlations among factors^§^.

	Mean (SD)	α	FSD	1	2	3	4
1. Perceived Risks	4.92 (1.24)	.936	0.973	-			
2. Perceived Usefulness	4.11 (1.11)	.863	0.954	-.284*	-		
3. Regulative Factors	4.18 (1.43)	.770	0.942	.306*	-.219*	-	
4. Normative Factors	3.84 (1.21)	.843	0.918	.310*	.543*	-.296*	-

^*§*^ SD = Standard deviation; α = Cronbach’s alpha coefficient; FSD = Factor score determinacy coefficient

* = p < .01.

No significant differences of mean scores of the four factors were found between groups of participants with different age and seniority. Significant differences were found for scores of Regulative Factors and Normative Factors, between gender and qualification. In particular, nurses showed higher scores for regulative factors (p = .001) and lower scores for normative factors (p < .001) than physicians; women showed higher scores for regulative factors (p = .006) and lower scores for normative factors (p < .001) than men. Results from GLMs showed that regulative factors and normative factors were significantly different (at p = .045 and < .001, respectively) only according to qualification (nurses vs physicians) after controlling for gender, age and seniority.

## Discussion

This study aimed to develop and psychometrically test the 28-item questionnaire ‘Digital Innovation Adoption in Hospitals’ (DIAH), a measure of institutional and individual determinants of WhatsApp usage in Italian hospitals [29]. The theoretical four-factor model was confirmed with acceptable fit indices. The correlations between factors were all significant. In particular, the high and positive correlation between ’Normative Factors’ and ’Perceived Usefulness’ is in line with the literature, where they appear as closely related [29]. Indeed, Normative Factors represent the levers that can be activated among peers in order to motivate the adoption of a new technology; in other words, the perceived usefulness of a new technology is shared between peers. The items in the section ‘Perceived Usefulness’ are designed in accordance with user acceptance models, which emphasize individuals’ rational and volitional assessment of the costs and benefits they would attain from the new technology [47]. According to these models, the use of a new technology is principally guided by a rational and voluntary individual’s choice, based on technology ‘acceptance’, which is strongly dependent on the nature of the technology (i.e. functionality and ease of use) [48]. The ‘Regulative factors’ explored the ‘adherence of nurses and physicians to the management’s objectives’, while the normative ones explore peer influence and patient influence. The regulative and normative factors are designed in accordance with organisational studies and theories, which conceive organisations as strongly institutionalised settings in which an individual’s behaviours are bounded by a complex combination of regulations, social norms and cultural systems [49, 50]. Regulative and Normative Factors were significantly different only based on qualification; the behaviours of nurses differed greatly from those of doctors. Indeed, doctors use WhatsApp more to communicate with patients and other team members, while nurses use it nearly exclusively for communication between nurses. Almost none of the involved nurses use WhatsApp to communicate with patients, only a few nurses report that patients ask them to use it to facilitate communication, and very few nurses suggest to patients to use WhatsApp. Moreover, since the effectiveness of WhatsApp for clinical education and for improving patients’ compliance has been demonstrated [13, 51] it would be important for nurses to exploit these results to improve patient education, which is one of the most important goals to achieve in nursing practice.

The section ‘perceived risks’ explored the following dimensions: safety for patients and healthcare professionals, safety in data sharing, data protection and clinical documentation, safety in clinical evaluation.

Beyond the validation of the questionnaire, the study also highlighted that WhatsApp was used in personal life and in the hospital environment by doctors and nurses in order to communicate and share data with patients and between peers [29]. In particular, its usage is mainly due to the perception of numerous advantages and benefits reported in clinical practice. However, healthcare professionals’ behaviours do not appear to be uniform. In fact, compared to doctors, nurses rarely use WhatsApp to communicate with patients or share clinical information among colleagues [29]. On the other hand, the use of WhatsApp is perceived to be unsafe for both patients and professionals, and its usage is inversely related to the perceived risk. At the same time, while nurses and physicians consider WhatsApp unsafe, they report using it anyway in clinical practice with both colleagues and patients, and a positive correlation emerged between ’Regulatory Factors’ and ’Perceived risks’. Finally, the study highlighted a negative correlation between ’Perceived Usefulness’ and ’Perceived Risks’, probably due to the fact that the more a tool is considered useful, the less the risks are perceived. This study presents some limitations that should be addressed by future research. A single-center survey was conducted with a relatively small number of participants, even if they were representatives of the main hospital wards and clinical areas. The number of participating nurses and women was higher with respect to the participating doctors and men. Moreover, the study investigated only nurses and doctors, thus excluding other health professions.

## Conclusions

The study tested the psychometric properties of the 28-item Italian ‘Digital Innovation Adoption in Hospitals’ (DIAH), a measure of the individual and institutional determinants of WhatsApp usage in hospitals. To our knowledge, this is the first tool available to measure which determinants influence healthcare professionals’ motivation to use WhatsApp in Italian hospitals. The results offer a novel insight for organization, research and practice.

For hospital organization, by combining organizational and individual factors in a coherent theoretical framework, the new questionnaire can help explore the connections between different determinants as well as their independent effects on the adoption of ‘employee-driven’ innovation. Moreover, the use of this new measure can help managers to oversee this phenomenon and to implement adequate strategies to exploit its potential increase, improving the safety both of patients and healthcare professionals.

This study represents a good starting point to frame the potential research that could be interesting to perform in the future. Further research should consider a multi-centre design to increase the generalizability of results, to explore the role that hospital characteristics–in terms of strategy, legacy, etc.–might have on shaping both the institutional and individual factors investigated in this study, and to improve the new theoretical framework proposed. Moreover, it would be interesting to create a specific questionnaire for each different profession and clinical setting, which include others health care workers beyond doctors and nurses. At the same time, to deepen current knowledge about this spreading phenomenon, it would be important to explore the determinants of WhatsApp usage through qualitative methods, such as interviews and focus groups.

From a practice point of view, the study represents an important starting point for policy makers, as it shed a new light on the importance to define guidelines for the safe use of such new digital technologies introduced by professionals without any formal evaluation. In fact, the results showed that, although hospital professionals consider WhatsApp not safe, nurses and physicians use this app in their clinical practice with both, colleagues and patients. For this reason, we assume that the use of WhatsApp in hospitals can be considered an extreme case of “back-door adoption”, and we point out the importance to define some guidelines for WhatsApp usage in the healthcare setting and in hospitals. Moreover, we think it could be relevant performing studies to explore the “perceived risks” and the reasons that lead healthcare professionals to use certain digital technologies even if they are perceived as not safe, and to evaluate the effectiveness of WhatsApp usage in different clinical areas.

As Pellegrino [52] wrote, “medicine is the most humane of sciences, the most empiric of arts, and the most scientific of humanities”, and the use of IT in clinical practice should be guided by such definition. This will only be possible if the new digital technologies in healthcare will increasingly be used based on proven scientific evidences and by referring to the guidelines of internationally recognized scientific societies. In this way hospital professionals and especially patients, who must always be the goal of every innovation in the hospital environment, would benefit from technological and digital improvements.

## Supporting information

S1 Dataset (ZIP)
